# Humic Substances Mitigate the Impact of Tritium on Luminous Marine Bacteria. Involvement of Reactive Oxygen Species

**DOI:** 10.3390/ijms21186783

**Published:** 2020-09-16

**Authors:** Tatiana V. Rozhko, Olga V. Kolesnik, Gennadii A. Badun, Devard I. Stom, Nadezhda S. Kudryasheva

**Affiliations:** 1 Krasnoyarsk State Medical Academy, 660022 Krasnoyarsk, Russia; 2 Institute of Biophysics SB RAS, Federal Research Center ‘Krasnoyarsk Science Center SB RAS’, 660036 Krasnoyarsk, Russia; olga.kolesnik.krsk@gmail.com (O.V.K.); n-qdr@yandex.ru (N.S.K.); 3 Department of Chemistry, Moscow State University, 119991 Moscow, Russia; badunga@yandex.ru; 4 Biology Department, Irkutsk State University, 664003 Irkutsk, Russia; stomd@mail.ru; 5 Biophysics Department, Siberian Federal University, Svobodny 79, 660041 Krasnoyarsk, Russia

**Keywords:** tritium, humic substances, luminous marine bacterium, bioassay, detoxification, reactive oxygen species, toxicity, adaptive response, hormesis

## Abstract

The paper studies the combined effects of beta-emitting radionuclide tritium and Humic Substances (HS) on the marine unicellular microorganism—luminous bacteria—under conditions of low-dose radiation exposures (<0.04 Gy). Tritium was used as a component of tritiated water. Bacterial luminescence intensity was considered as a tested physiological parameter. The bioluminescence response of the marine bacteria to tritium corresponded to the “hormesis” model: it included stages of bioluminescence inhibition and activation, as well as the absence of the effect. HS were shown to decrease the inhibition and activation effects of tritium, similar to those of americium-241, alpha-emitting radionuclide, studied earlier. Correlations between the bioluminescence intensity and the content of Reactive Oxygen Species (ROS) were found in the radioactive bacterial suspensions. The results demonstrate an important role of HS in natural processes in the regions of low radioactive contamination: HS can mitigate radiotoxic effects and adaptive response of microorganisms to low-dose radioactive exposures. The involvement of ROS in these processes was demonstrated.

## 1. Introduction

Low-intensity radioactive contaminations create problems in the vast territories of the world. Low-intensity radiation is characterized by the concentration of radionuclides of alpha- and beta-type or/and dose rate of gamma rays which produce a “low-dose” impact on organisms. According to [1], a tentative limit of low doses for high organisms is 0.1 Gy. In general, toxicology suggests that the low-dose border separates the low-dose effects from toxic effects: the latter means the suppression of physiological functions of organisms by radiation. However, this limit can change under variation of the environmental conditions and the state of an organism [2,3]. The sensitivity of organisms to the low-dose radiation impact is not entirely clear yet; the variation of the sensitivity under natural conditions is a question of special interest.

It is known that biological responses to irradiation can change under a variety of molecular surroundings [2,3]. The addition of poly-functional organic molecules can change ionic and radical states of water solutions of alpha- and beta-emitting isotopes, thus changing the surrounding of water inhabitants and hence, their responses to the radionuclide solutions. Unicellular organisms are the proper tools for studying biological responses in complex media. Microbiota of the aqueous media might be sensitive to the presence of humic substances (HS), products of natural decomposition of organic matter, which play a role of natural attenuators of environmental toxicity [4,5,6,7,8] due to their complexing and redox ability. The presence of phenolic and other redox-active functional groups in HS accounts for their capability of reducing toxic impacts of organic and inorganic oxidizers [9,10,11], similar to the redox activity of other natural phenolic compounds [12,13]. The biological activity of HS is intensively discussed now [14,15,16,17,18,19].

Molecular mechanisms of biological responses to low-intensity radiation in the presence of HS are of practical interest; they allow for the prediction of the response of living organisms to low-intensity radiation in large territories after nuclear accidents, discharges of nuclear plants, or underground mining.

The luminous marine bacterium is a proper bioassay system for radiotoxicity monitoring in the complex multicomponent media. This bioassay is classic [20,21]; it applies bioluminescence intensity as a physiological testing parameter. The advantages of the bioassay are high sensitivity, simplicity, high rates of the assay procedure (1–3 min), and availability of devices for the toxicity registration. These advantages provide the possibility of numerous sample analyses and proper statistical processing. This is highly important for low-dose effects which are known to be characterized as stochastic [22,23].

**Figure 1:**
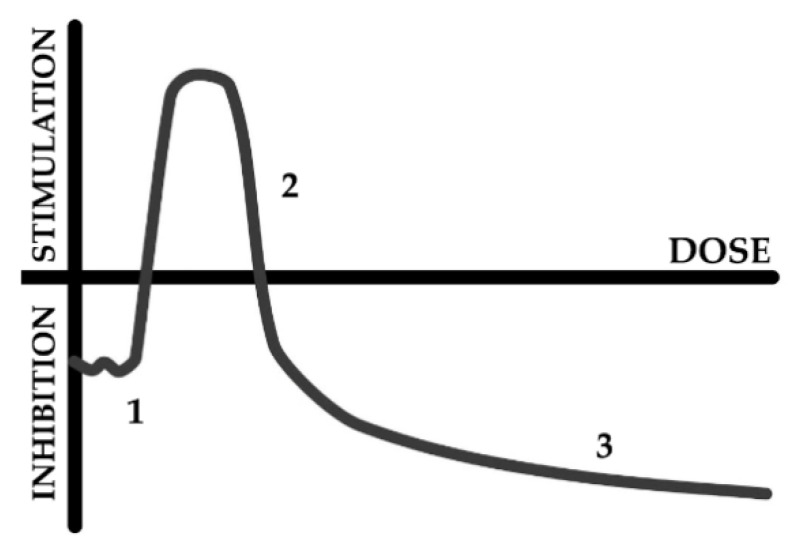
Scheme of hormesis dose–response model. The response includes stages of stress recognition (1), stimulation (activation) (2), and inhibition (3).

Our previous works revealed the peculiarities of the response of luminous marine bacteria to the low-dose radiation of alpha-, beta-, and gamma-types [15,24,25,26,27,28,29,30,31]. Bacterial responses to alpha- and beta- radionuclides corresponded to the conventional “hormesis” dose–response model [32,33,34,35]. The scheme of this model is presented in Figure 1; it includes three stages: stress recognition, stimulation (activation), and inhibition of an organismal function. 

Previous investigations [27,36] analyzed the effect of HS on luminous marine bacteria exposed to alpha-emitting radionuclide americium-241. The influence of americium-241 corresponded to the hormesis model: the activation and inhibition stages were evident, while the first stage (adaptive response) manifested itself as an induction period. The addition of HS did not change a type of the kinetic curve, but changed the bioluminescence intensity, decreasing deviations of the bacterial luminescence intensity from the control (non-irradiative) sample. Hence, the mitigation of the bioluminescence response to alpha-emitting radionuclide americium-241 was observed.

A comparison of HS mitigation effects in solutions of alpha and beta radionuclides is a topic of our special interest. Both radiation types, alpha and beta, are of corpuscular nature but differ in their bioeffects. Beta particles are characterized by low energy, size, and high initial speed; they have higher penetrative power, but hundreds of times less ionization ability as compared to the alpha particles. Beta particles can partially penetrate into living tissues, and transfer energy to cells and their components. 

In the current study, we chose tritium as a source of beta radiation. The choice was justified by an environmental prevalence of tritium; it is one of the most common decay products in the nuclear industry. Cases of activation and inhibition of bacterial bioluminescence by tritium were under consideration in [15,22,28,29,30,31]. The effects of tritium on bacterial enzymes were studied as well [30]. The application of bacterial enzymes as bioluminescence assay systems was developed earlier [37,38,39,40,41].

Tritium occurs mainly as a component of tritiated water, HTO; it contributes to the natural background in water bodies as 2 Bq/L. The native content of tritium is not dangerous for organisms. High-energy tritium exposures (>4500 GBq or >124 Ci) are toxic for organisms suppressing their physiological functions [42,43]. The high-energy chronic toxicity of tritium is concerned with its ability to substitute protium, a naturally prevailing hydrogen isotope, in organic compounds. Radioactive decay transforms tritium to an ionized isotope of helium-3, H23e+, with the emission of electron and antineutrino:

H13→ β− H23e++e−+ν˜

This transformation is followed by the destruction of tritium-substituted organic compounds and the rupture of intermolecular hydrogen bonds. In the case of biological macromolecules, such as DNA, enzymes and coenzymes, the tritium decay results in disruption of genetic, epigenetic, and biochemical processes in cells.

The average energy of tritium beta particles is low (5.69 keV), but the ionization ability of tritium in water media is high. Under low-dose conditions, the charged products of tritium decay (H23e+ and electron) affect the membrane functions and trigger charge transfer in intracellular processes. 

Products of radioactive decay of HTO-molecules are electrons and Reactive Oxygen Species (ROS):

*H*–*O*–*T* → *H*–*O*–^3^*He*^+^ + *ē*

*H*–*O*–^3^*He*^+^ → *H*–*O*^.+^ + ^3^*He*

*H*–*O*^+^ + *H*_2_*O* → [*O*] +^.^*H*_3_*O*^+^

where [*O*] is atomic oxygen, representative of ROS. 

ROS are a group of reactive compounds, products of partial reduction of oxygen. The ROS group includes superoxide anion (*O*_2_•−), hydroxyl radicals (*OH*•), hydrogen peroxide (*H*_2_*O*_2_), and others [44]. ROS are known to be generated in water solutions under radiation exposures in the presence of dissolved molecular oxygen [24,45,46,47]. On the other hand, ROS are products of native metabolic oxidative reactions [44,48]. For example, results of Rozhko and co-authors [22] demonstrated that marine bacteria naturally increase ROS content in aquatic media, and they intensify the ROS production at the addition of HTO.

According to the modern approaches, ROS can react both as damaging and signal agents [49,50]; they regulate organismal functions, such as apoptosis or protective responses in cells [46]. ROS are responsible for proliferation, migration, differentiation, and metabolism [51,52]; they are considered as stimulators of a cell division [53,54] or cell death—apoptosis, autophagy and necrosis [44,55,56]. The signal function of ROS is intensively discussed now [57,58,59]; a pioneer in this approach was Proctor [54]. It should be noted that ROS can serve both as inter- or intracellular messengers [60,61,62]. Reactive oxygen or nitrogen [63] species released by cells can serve as signal particles which elicit the radiation-induced bystander effect [64,65].

Interrelations between the ROS content and organismal physiological functions can elucidate the molecular mechanism of the protective response to the beta radiation. Correlations between the ROS content and bacterial bioluminescence intensity in HTO were demonstrated in [22], thus revealing the involvement of ROS in the activation of the bacterial physiological function in the radioactive solutions. In this paper, we described (1) changes of the bacterial response to tritium under the addition of HS, and (2) variation the ROS content in radioactive bacterial suspensions at HS addition. The involvement of ROS in the mitigation of tritium effects on luminous marine bacteria is of interest.

## 2. Results and Discussion

To analyze the HS impact on the bacteria in tritiated water, HTO, we considered the different type effects of HTO on the bacterial luminescence—inhibition, activation and the absence of the influence. 

According to the hormesis toxicological model [32,33,34,35], Figure 1, the stages of the dose–response model demonstrate either activation or inhibition of organismal physiological functions; the absence of the effect can occur as well. It should noted that our previous studies revealed variations in the bacterial luminescence response to tritium in the frames of hormesis model: bi-phasic time dependence (activation + inhibition) was found in [30,31], and the mono-phasic time dependence (only activation) was found in [22,28,29]. In addition, variations of the effect types were observed under the conditions of low-dose alpha and gamma irradiation [25,26,27,31]. All these variations are explained in terms of the “hormesis” approach [32,33,34,35]: the organismal response is not unified; it depends on the type of organism, its state, and external conditions; all the response specificities are the particular cases of the generalized hormesis model. Additionally, it is known that a dose–response dependence can be absent at all, the low-dose radiobiological responses can be “noisy”. The absence of the dose–response dependency corresponds to a concept on “stochasticity” of radiobiological low-dose effects [66]. This concept suggests the involvement of ROS and other free radicals to a radiobiological response.

### 2.1. Mitigation of the Bacterial Luminescence Response to Tritium by Humic Substances

**Figure 2:**
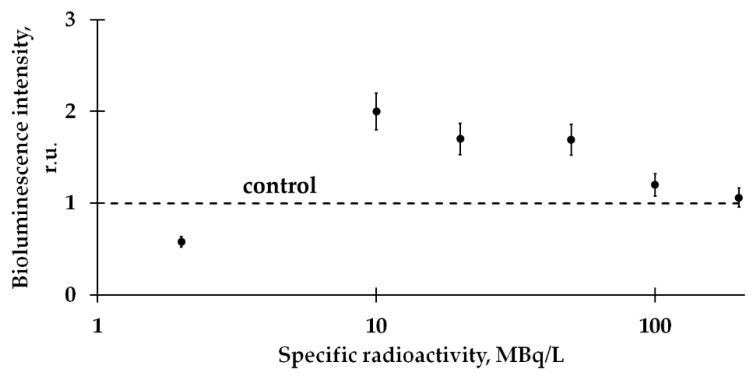
Relative bioluminescence intensity at different specific radioactivities of tritiated water (HTO), 6-h exposure time. Values of the specific radioactivities for the samples used are listed in Section 3.2.1.

**Figure 3:**
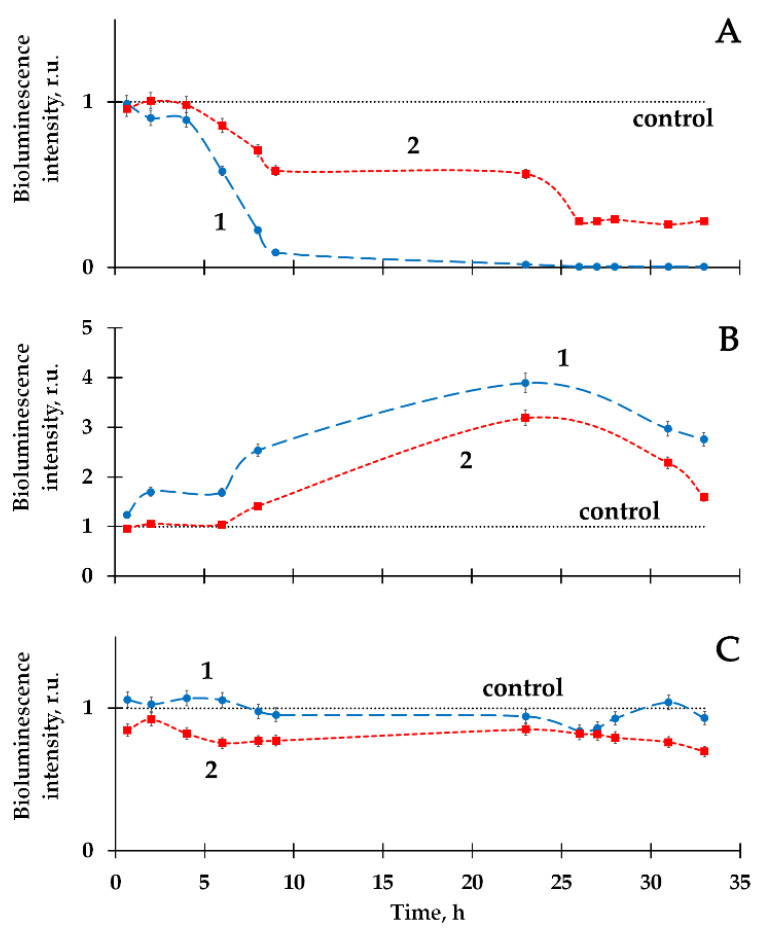
Bacterial bioluminescence kinetics in HTO in the absence (1) and presence (2) of humic substances (HS). Specific radioactivity of HTO: (**A**) 2 MBq/L; (**B**) 50 MBq/L; (**C**) 200 MBq/L. HS concentration—10^−3^ g/L.

Preliminarily*,* the dependence of the bacterial luminescence on HTO activity concentration (specific radioactivity) was studied; kinetic bioluminescence curves were measured under the exposure to HTO. The bioluminescence intensity at different HTO activity concentrations is presented in Figure 2; examples of the bioluminescence kinetic curves in radioactive solutions are presented in Figure 3 (curve 1). In the course of our experiments, we did not exceed a tentative limit of low-dose tritium effects: the maximal dose accumulated by the bacterial suspensions was 0.04 Gy.

Figure 2 demonstrates that the effect of HTO on the marine bacteria preparation corresponds to the first and second stages of the hormesis model, Figure 1. To provide the following experiments, we chose different types of HTO effects (inhibition, activation and absence of the effect) at different HTO radioactivities (2, 50 and 200 MBq/l, respectively). Bioluminescence kinetic curves of these samples are presented in Figure 3A–C (curve 1), respectively. These curves reveal deviations of the bioluminescence intensity in radioactive solutions from this in the non-radioactive (control) ones.

The influence of humic substances, HS, on the bioluminescence kinetics was analyzed. The bioluminescence response in the solution: bacteria+HS+HTO was compared to this in the solution: bacteria+HS (control sample). The values of the relative bioluminescence intensity are presented in Figure 3A–C (curve 2). As demonstrated previously [11,67], HS could increase the bacterial bioluminescence up to 40% at concentrations <10^−1^ g/L; no monotonic dependence on HS concentration was found. The effect of bioluminescence activation has been taken into consideration by using bacteria+HS samples as control.

Curves 1 and 2 compare the effects of HTO in the absence and presence of HS. It is seen that HS move the kinetic curves closer to the control for bioluminescence inhibition (Figure 3A) and activation (Figure 3B), mitigating the HTO impact. Figure 3C demonstrates that HS change the bioluminescence kinetics negligibly in the case of the absence of an initial HTO impact.

It is likely that the mitigating effect of HS on the system bacteria+HTO can be concerned with HS polyfunctionality. Since the HS macromolecules involve a lot of electron–donor and electron–acceptor functional groups, they can act as a kind of “buffer” in the electron-transfer processes and moderate the deficiency or excess of unstable charged/radical particles in water solutions. These changes might moderate the cellular processes via the intermediation of the cellular membranes. 

ROS content in the bacterial suspensions might be sensitive to the presence of HS, too. In [22], the low-dose effect of tritium (<0.08 Gy) on luminous marine bacteria was proved to be concerned with the intensification of ROS production by the bacteria. It was demonstrated that a rise of ROS concentration in the bacterial suspensions correlated with the intensification of the bioluminescence in the bacteria during the bacterial lifetime. The low-dose effects of HTO were explained with “bystander effect” in the bacterial suspension based on the involvement of ROS as intercellular messengers.

It is likely that the mechanism of HS mitigation of the bioluminescence response at low-dose exposure to tritium is concerned with HS involvement in ROS production by the bacteria. To verify this assumption, we analyzed ROS content in the control and radioactive bacterial suspensions in the presence and absence of HS.

### 2.2. Variation of ROS Content by Humic Substances in Radioactive Bacterial Suspensions 

**Figure 4:**
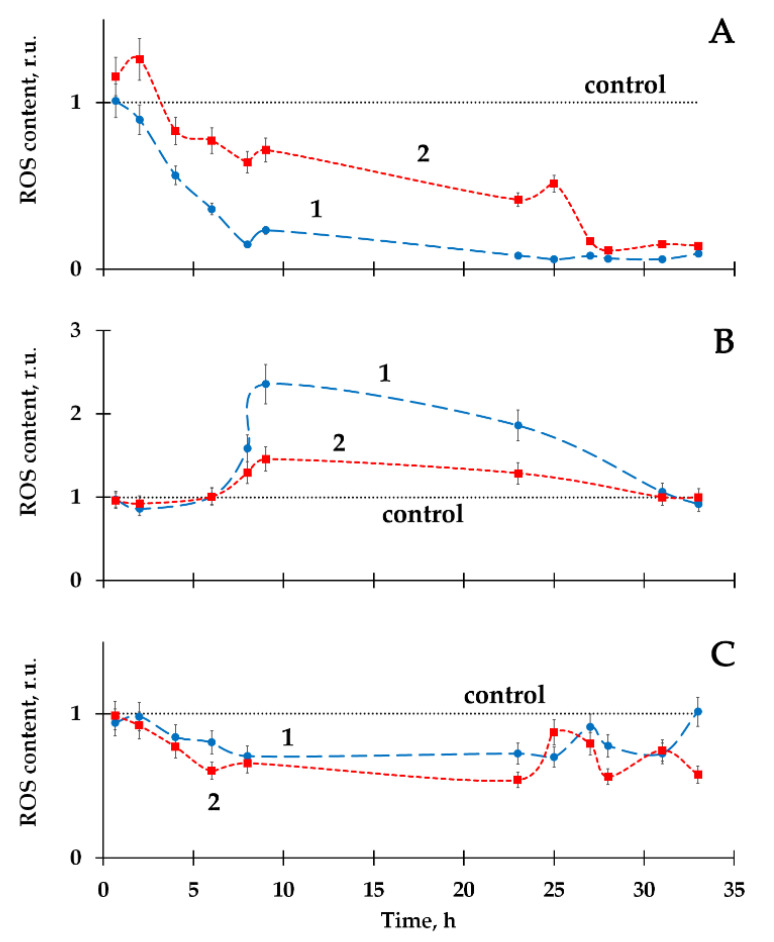
Reactive oxygen species (ROS) content in the bacterial suspensions in HTO in the absence (1) and presence (2) of HS. Specific radioactivities of HTO: (**A**) 2 MBq/L; (**B**) 50 MBq/L; (**C**) 200 MBq/L. HS concentration—10^−3^ g/L. ROS content in the control sample—10^−6^M.

The ROS content was monitored in a course of the experimental bacterial lifetime using the chemiluminescence luminol method. Figure 4A–C show the results of ROS measurements in the bacterial suspensions of different radioactivity in the absence (curve 1) and presence (curve 2) of HS.

The decrease (Figure 4A, curve 1) and increase (Figure 4B, curve 1) in ROS content were observed in the radioactive bacterial suspensions, as compared to the control (non-radioactive) samples.

It should be noted that the time-courses of ROS content in bacterial suspensions+HTO was studied in detail in [22]. Only ROS increase by HTO was reported there. Current experiments demonstrate the variation in the bacterial responses, being closer to the classic type of hormesis curve (Figure 1). It is likely that a decrease in ROS content in bacterial suspensions at low specific radioactivity (2 MBq/L, Figure 4A) might be explained by the intensive ROS consumption in the bacterial bioluminescence reaction catalyzed by bacterial luciferase, followed by the formation of the intermediate of this reaction—peroxide derivative of flavin [40,68]. The increase in ROS content in the bacterial suspensions at higher specific radioactivity (50 MBq/L, Figure 4B) can be attributed to the intensification of the complex metabolic processes on the bacterial cells as a response to the radioactive impact of tritium, similar to the explanation presented in [22].

Figure 4 shows that HS moved the ROS-content kinetic curves closer to the control (curve 2, Figure 4A,B). Similar to the HS influence on the bacterial bioluminescence (Section 2.1), these effects might be explained by polyfunctionality of HS macromolecules and a tendency to reversible electron acceptation by HS resulted in the regulation of the content of radical oxygen-containing particles.

Figure 4C presents the absence of HS influence on the ROS content. 

The similarity of the time-courses of the ROS content and bioluminescence intensity in the radioactive suspensions is evident (comparing Figure 3 and Figure 4). The results suggest the involvement of HS to the regulation of ROS content in the bacterial suspensions, followed by the variations in the bacterial bioluminescence intensity.

To confirm the relations between the bacterial bioluminescence and the ROS content in the bacterial suspensions, the correlation coefficients *R* were calculated and presented in Table 1. 

The table shows that high positive correlations between the bacterial bioluminescence yields and the ROS contents were observed in the suspensions of luminous bacteria in the presence and absence of HS.

As an outline, it is shown that HS change the effects of the radionuclide tritium on the luminous marine bacteria through the intermediation of ROS.

## 3. Materials and Methods

### 3.1. Preparations and Reagents for Bioluminescence and Chemiluminescence Measurements

Cellular bioluminescence assay was used to monitor the effects of HTO in the presence and absence of HS. The assay was based on lyophilized *Photobacterium phosphoreum* which was produced by a standard technique [69]. The strain was obtained from the Collection of Luminous Bacteria CCIBSO-836, Institute of Biophysics SB RAS, Krasnoyarsk, Russia. The NaCl preparation (chemical grade, Khimreactiv, Staryy Oskol, Russia) was applied to prepare the 3% solution for the bacterial bioluminescence measurements.

Reagents for the chemiluminescence measurements were: luminol from Sigma-Aldrich, hydrogen peroxide solution, H_2_O_2_, from Tula Pharmaceutical Factory, Tula, Russia, K_3_[Fe(CN)_6_] from Khimreaktiv, Russia. The reagents were of chemical grade.

The Gumat-80 preparation, “Gumat”, Irkutsk, Russia, produced by non-extracting treatment of coal [70] was used as a source of HS. The HS concentration chosen was 10^−3^ g/L; it was based on the HS effects on the bacterial luminescence studied in [11,67].

### 3.2. Bioluminescence Measurements

#### 3.2.1. Bacterial Bioluminescence Samples

The bacterial suspension samples were prepared from lyophilized bacterium preparations according to standard technique [69]: to imitate a marine environment for the bacterial cells and to balance osmotic processes, the 3% NaCl solutions were used; final bioluminescence intensity of the bacterial suspension samples was controlled. Tritiated water, HTO, JSC Isotope, Russia, was used as a source of tritium. HTO was added to NaCl solutions and mixed with the bacterial suspensions to the final specific radioactivities: 2, 10, 20, 50, 100, and 200 MBq/L.

Bioluminescence kinetics was studied in the bacterial suspensions of different compositions: bacteria; bacteria+HS; bacteria+HTO; bacteria+HTO+HS. The bacterial suspensions were prepared as follows: control samples: 400 μL of non-radioactive bacteria suspensions were added to 1600 μL of 3% NaCl solution. Samples bacteria+HS: 400 μL of bacterial suspensions and 200 μL of HS were added to 1400 μL of 3% NaCl solution. Radioactive samples bacteria+HTO: 400 μL of bacterial suspensions were added to 50 μL of HTO in 1550 μL of 3% NaCl solution. Radioactive samples bacteria+HTO+HS: 400 μL of bacterial suspensions and 200 μL of HS were added to 50 μL of HTO in 1350 μL of 3% NaCl solution. 

#### 3.2.2. Bioluminescence Registration

To investigate the chronic effects of the low-level beta radiation of tritium on the bacterial bioluminescence, a standard procedure for the bioluminescence measurements was used [22,69]. Bioluminescence intensities of the control and radioactive samples were measured and compared in the presence and absence of HS, the relative bioluminescence intensities were calculated as ratios of bioluminescence intensities in solutions: bacteria+HTO vs. bacteria, and bacteria+HTO+HS vs. bacteria+HS. All bioluminescence measurements were carried out in four replications; the experimental error did not exceed 5%.

Bioluminescence intensity was registered by Luminoskan Ascent (Thermal Fisher Corp., Waltham, MA, USA). All measurements were carried out at +20 °C as the room temperature provides the maximal activity of luminous marine bacteria [20,21].

### 3.3. Chemiluminescence Measurements

The luminol chemiluminescence method was used to evaluate the ROS content in the experimental bacterial suspensions. This method is classic [71,72]; it determines an integral content of ROS assuming that a mobile equilibrium of the different ROS forms takes place. Additionally, this method is highly convenient in a complex application with bioluminescence measurements, as these two methods use the same instrumentation for the light-emitting registration. The chemiluminescence registration was carried out just after the bioluminescence measurements in the same bacterial samples.

The calibration dependence was preliminarily determined as a chemiluminescence intensity vs. H_2_O_2_ concentration; H_2_O_2_ was applied here as a ROS model. Concentrations of alkaline luminol and K_3_[Fe(CN)_6_] solutions were 10^−4^ M and 10^−3^ M, respectively.

Maximal chemiluminescence intensity was determined in all bacterial suspensions (see Section 3.2.1) after the bioluminescence measurements. First, the luminol solution was added to the bacterial samples. Then, the chemiluminescence reaction was initiated by 75 μL solution of K_3_[Fe(CN)_6_] through the injection system. The experimental error of the chemiluminescence measurements did not exceed 10%.

Chemiluminescence intensity was registered by Luminoskan Ascent (Thermal Fisher Corp.). All measurements were carried out at room temperature.

### 3.4. Statistical Processing

To evaluate correlations between the bioluminescence signal and ROS content, Pearson’s correlation analysis was applied [73]. Statistical dependence between the rankings of two variables was analyzed: correlation coefficients *R* were calculated between the bioluminescence and chemiluminescence quantum yields at different HTO specific radioactivities—0, 2.0, 10, 20, 50, 200 MBq/L.

The relative quantum yields were evaluated as integral values over the periods of the bioluminescence observation. The application of the method was justified by the normality of the data distribution at different HTO concentrations. Verification of the normality was carried out using D’Agostino–Pearson normality test and Kolmogorov–Smirnov test [74].

## 4. Conclusions

We confirmed in this paper that a response of marine bacteria to the beta-emitting radionuclide tritium corresponds to a hormesis model: it includes stages of inhibition and activation. Correlations between the bioluminescence yields and ROS content were revealed, confirming the involvement of ROS to the low-dose effect of tritium. Following our previous results [22], we explain the low-dose effects of tritium by the “bystander effect” based on the signaling role of ROS in the bacterial suspension.

Humic substances were shown to decrease both inhibition and activation effects of tritium on the luminous bacteria. Similar effects of HS on bacterial luminescence were previously observed for the alpha-emitting radionuclide—americium-241 [27]. The result demonstrates an important role of humic substances in natural processes in the regions of low radioactive contamination: humic substances can mitigate the radiotoxic effect and adaptive response of microorganisms to low-dose radioactive exposures. The involvement of ROS in these processes was demonstrated, with the beta-emitting radionuclide tritium as an example.

## Figures and Tables

**Table 1:** Correlation coefficients *R* between the bacterial bioluminescence yields and ROS contents in the bacterial suspensions.

*R*	
without HS	in the presence of HS
0.99	0.95

## Abbreviations

HS	humic substances
HTO	tritiated water
ROS	reactive oxygen species
*R*	Pearson correlation coefficient
